# Mammalian cycles: internally defined periods and interaction-driven amplitudes

**DOI:** 10.7717/peerj.1180

**Published:** 2015-08-13

**Authors:** LR Ginzburg, CJ Krebs

**Affiliations:** 1 Department of Ecology and Evolution, Stony Brook University, Stony Brook, NY, USA; 2 Department of Zoology, University of British Columbia, Vancouver, BC, Canada

**Keywords:** Lynx-hare cycle, Population cycles, Maternal effect, Population dynamics, Eigenperiod hypothesis, Amplitude and period

## Abstract

The cause of mammalian cycles—the rise and fall of populations over a predictable period of time—has remained controversial since these patterns were first observed over a century ago. In spite of extensive work on observable mammalian cycles, the field has remained divided upon what the true cause is, with a majority of opinions attributing it to either predation or to intra-species mechanisms. Here we unite the eigenperiod hypothesis, which describes an internal, maternal effect-based mechanism to explain the cycles’ periods with a recent generalization explaining the amplitude of snowshoe hare cycles in northwestern North America based on initial predator abundance. By explaining the period and the amplitude of the cycle with separate mechanisms, a unified and consistent view of the causation of cycles is reached. Based on our suggested theory, we forecast the next snowshoe hare cycle (predicted peak in 2016) to be of extraordinarily low amplitude.

## Introduction

**Figure 1:**
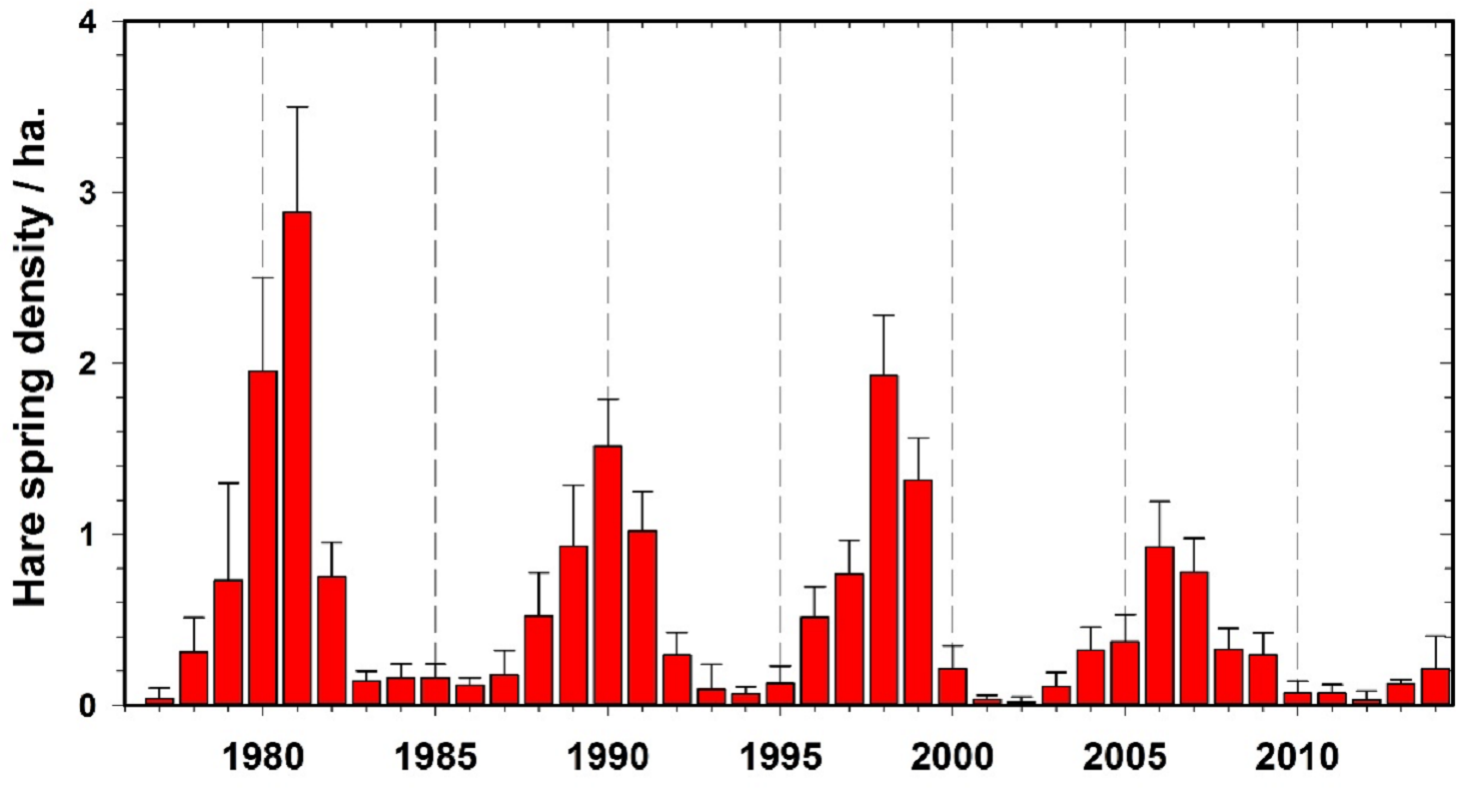
Observed hare density cycles for the period of 1975–2014, Kluane Lake, Yukon, with 95% confidence limits.

The cause of mammalian cycles—the rise and fall of populations over a predictable period of time—has remained controversial since these patterns were first observed over a century ago. The intractability of this problem is surprising given that biologists can often observe many cycles (of rodents or hares, for example) in one human lifetime. (Dambacher et al., 1999; Krebs, 2011; White, 2013; Fig. 1) In spite of extensive work on observable mammalian cycles, the field has remained divided upon what the dominant cause is, with a variety of mechanisms postulated. A majority of opinions on the causes of cycles attribute them to either predation or to internal effects. An ‘either-or’ confusion over these two effects is unwarranted, and we postulate here that a ‘both-and’ set of explanations would further research designs and reduce controversy.

In a closely related area of biology, host-parasite interaction, a similar division of views has been prevalent. The recent review of the situation (Turner et al., 2014, p. 1007) describes it as “vicious circles: synergy between condition and infection.” Parasites are more likely to infect weaker hosts and they influence the host dynamics. Whether hosts have an additional “cause of decline” which is exacerbated by parasites is the question. The well-known experiments by Hudson, Dobson & Newborn (1998) suggested a reduction of cyclicity of red grouse by artificially reducing the parasite load. It was, however, noted by Tompkins & Begon (1999) that the period of the cycle seemed to remain the same while the amplitude was apparently reduced. Begon, Townsend & Harper (2006) have reiterated this interpretation, where they say, “…The results are also consistent with the nematodes determining the amplitude of the cycles, but not generating them in the first place.” Krebs’ well-known field experiment (Krebs et al., 1995) isolating hares from lynx and coyotes also have shown the same period but a much higher amplitude. No experiment so far has been able to abruptly stop a population cycle, and if the mechanism affecting the amplitude was manipulated effectively, then by our hypothesis the period of the oscillations would not be affected. Therefore, whether predators and parasites are “passengers and not drivers of the cycle” as suggested by White (2013) depends on whether the cause of the cycle is identified with the cause of the period or the cause of the amplitude. Our view, in full agreement with Moss & Watson (2001), is that the two aspects can often have different causes and predators are passengers from the period point of view, they inherit the period of the cycling prey, if the prey cycles. Prey, however, may not cycle at all without the appropriate disturbance and the very existence of cyclicity, not just its period, may depend on predator,parasite or social influences.

When periods of cycles are low, an interesting recent work by Barranquand et al. (2014) suggested that the much simpler, first order density dependent process may serve as an explanation and that two-dimensional dynamics is not necessary. However, we think that for long periods the delayed density dependence has to be invoked. Maternal effect is not the only mechanism to achieve it, but it has some nice properties which make us focus on it as an explanation for the period (see Eigenperiod hypothesis below).

We will attempt to show that trying to choose between the internal and external causes is the root of the difficulty in finding a satisfying explanation. By explaining the period and the amplitude of the cycle with separate mechanisms a unified and consistent view of the causation of cycles can be reached. Here in one example we unite the eigenperiod hypothesis of Ginzburg which describes an internal, maternal effect-based mechanism to explain the cycles’ period, with Krebs’ recent generalization explaining the amplitude of snowshoe hare cycles based on initial predator abundance (Krebs et al., 2014).

## Discussion

### Eigenperiod hypothesis

According to the maternal effect model (Ginzburg & Colyvan, 2004, p. 59), every population has a tendency to oscillate in abundance with an intrinsic period. The period varies inversely with the *R*_max_ value (Ginzburg & Colyvan, 2004, p. 53). In Krebs et al.’s (1986) data, the cycle period of hare oscillations declines from 10 to 8 years when *R*_max_ goes up from 2.0 to 3.0 and even 4.0, consistent with the proposed mechanism. This does not mean that every population has to undergo periodic oscillatory behavior. In order to cycle, the population has to be appropriately disturbed.

Let us elaborate on this with a physical analogy. A suspension bridge or a guitar string is stationary most of the time, but if appropriately disturbed, it oscillates with its own so-called natural frequency. Physicists use the word *natural* for this frequency, stressing that it is a property that belongs to the bridge or the guitar string and not to the way in which they are disturbed. In biology, the word *natural* has so many meanings that we have decided to use the more technical but unambiguous terms *eigenfrequency* and *eigenperiod*. An eigenfrequency is an intrinsic property of the species—its tendency, if appropriately disturbed, to oscillate at a fixed frequency. Amplitude and the shape of the oscillation that a bridge may undergo do depend on the disturbance; it is only the period that does not. The cause of this simple and clear separation is second-order dynamics that are basic in the world of mechanical objects. Likewise, our view is that for vertebrates maternal effects with density dependence result in fundamental second-order dynamics for the single species and it is this fact that explains the eigenperiod.

The problem of explaining cyclicity of snowshoe hares in North America or cyclicity of voles and lemmings in northern Europe has been under study for more than 75 years, without a clear resolution (Krebs et al., 2001). Moreover, well-known experiments (Krebs et al., 1995; Krebs, Boutin & Boonstra, 2001) failed to tame the controversy (e.g., White, 2013). Our argument is able to provide a natural explanation for why similar species cycle with similar periods. For example, we know that hares cycle with a period of around 10 years when lynx are a major predator, but the period is the same on islands where foxes are the major predator (Peterson & Vucetich, 2002) or when great-horned owls are a major predator (Elton & Nicholson, 1942). We suggest that the period of 10 years is an eigenperiod for hares.

The 4-year cycles of voles and lemmings (about 12 generations long in this case) may also be the eigenperiod for these species (Inchausti & Ginzburg, 1998). Lemmings and voles cycle with similar period even in the absence of predators (Menyushina et al., 2012). What is the chance that these species exhibit consistent periods in drastically different ecosystems, from tundra to temperate grasslands, and with and without predators, if the period is determined by the predator–prey interaction? We believe that this is unlikely indeed.

Our view includes the absence of cyclicity, which is what is seen most of the time for most species. A pendulum placed in a viscous liquid will not oscillate. Likewise, strong, direct density dependence (acting like friction) will stabilize population abundances. Boonstra (1994) gives an extensive discussion of the reasons for noncyclicity. One needs a combination of low direct density dependence and the right kind of disturbance to cause observable cyclicity. Given that this combination is present, our suggestion is that the period of oscillation is an eigenperiod.

Maternal effects, in which energetic quality is passed from mothers to offspring, have been known to be a potential cause of cycles since Wellington (1957). They have been a research focus in mammal ecology since the work of Boonstra & Boag (1987) on voles and an exposition by Rossiter (1991). Ginzburg & Taneyhill (1994) described the first mathematical model combining density dependence and a delay due to internal quantity-quality dynamics.

The argument that maternal effects explains the period of population cycles is based on observations about the lengths of cycles when properly expressed in units of generation times of the cycling prey species, rather than in chronological units (Ginzburg & Colyvan, 2004). The period is predicted theoretically to always be greater than 6 generations and to decline with increasing values of *R*_max_. For typical *R*_max_ values of about 2.0 per generation, the period is predicted to be about 7–8 generations, which is consistent with the evidence (Ginzburg & Colyvan, 2004). Calder (1983), followed by Peterson, Page & Dodge (1984) and Krukonis & Schaffer (1991), have found a clear allometry between cycle period and prey body size in all cycling species. The slope of the allometric relationship corresponds to 6–7 generations on average. At the same time the allometry of the cycle period with predator body size is flat, suggesting that the prey population drives the cycle period. It forms a wave travelling up the trophic chain in which predators lag (by their gestation time) behind the abundance of their food.

In a little known paper, Bulmer (1975) developed a time-delay test to determine whether predator–prey joint dynamics is a single cause of the cycle or not. Using the most parsimonious interpretation of the Bulmer model, we conclude that if predator abundance is simply following the food availability, the delay will be equal to the gestation time of the predator. If the predator is actually the cause of the prey-decline portion of the cycle, the delay has to exceed 1/4 of the period. Bulmer immediately concluded that the lynx-hare sequence does not have a sufficient time delay to be explained by predation alone. In a re-analysis, Inchausti & Ginzburg (2002) observed a small delay, but much less than 1/4 period. Data from the recent study by Krebs et al. (2014) show a small one-year time delay of the lynx abundance after hare abundance, a strong argument in favor of an internal mechanism in the hare.

### Predator defined amplitude hypothesis

**Figure 2:**
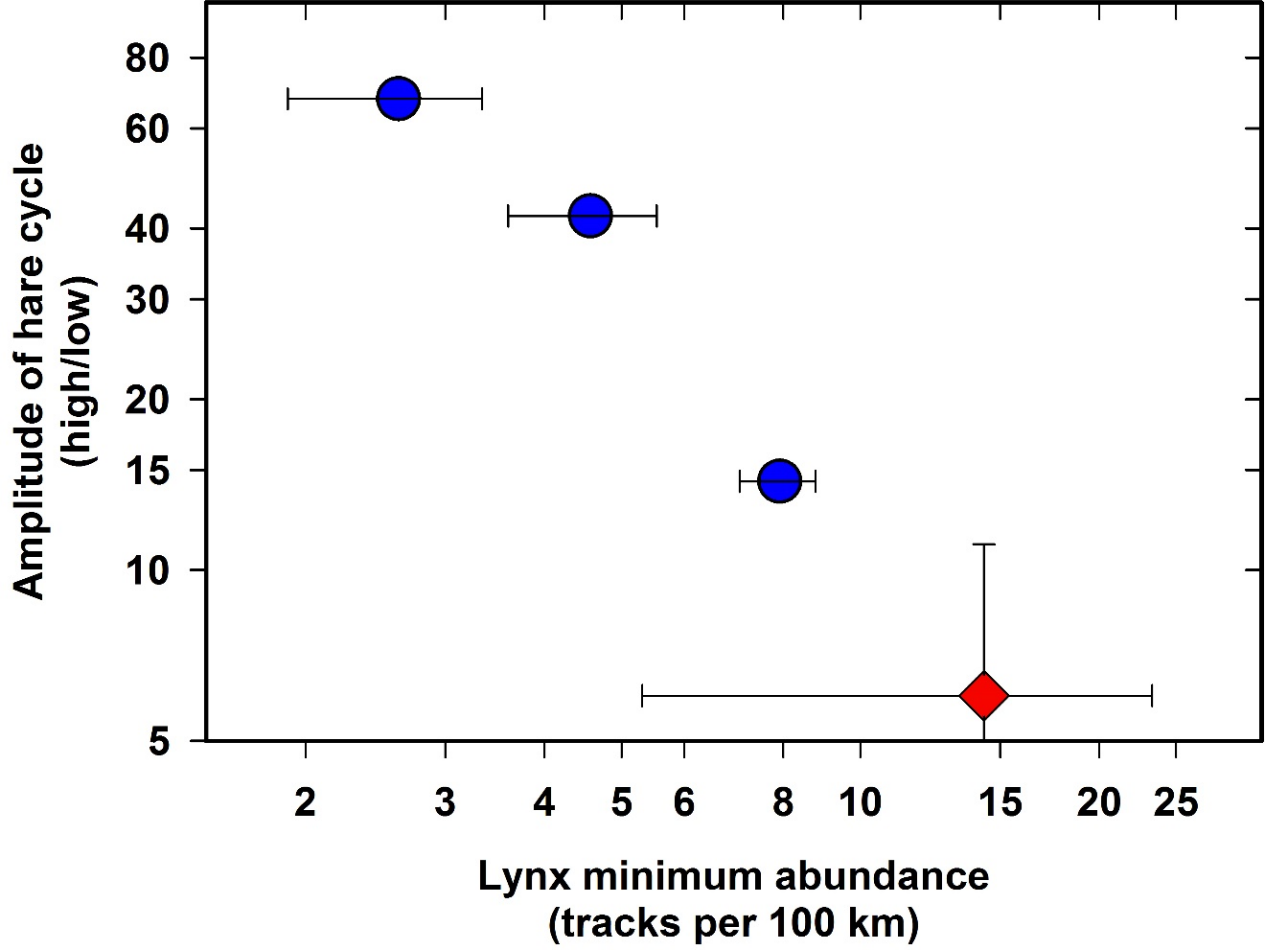
Minimum abundance of lynx at the point where the hare cycle enters its increase phase in relation to the measured amplitude of three snowshoe hare cycles, Kluane Lake, Yukon, 1987–2012. Error bars are 1 S.E. During the first of the four cycles shown on Fig. 1, lynx abundance was not tracked. The lowest amplitude data point (marked in red) is our 2014 prediction for the ongoing cycle: we forecast the lowest observed amplitude of 6. Poor snow conditions restricted sampling for lynx snow tracks in the most recent low phase (2012), resulting in large error bars for lynx minimal abundance and for the predicted next hare peak.

A recent review by Krebs et al. (2014) listed four hypotheses as mechanisms for the variation in amplitude of snowshoe hare cycles in northwestern North America—weather, forest succession, plant defense, and predation. The most parsimonious of the four with our present data is the predator hypothesis. The observation is that predator lows (indexed by lynx abundance prior to the start of the cycle) are inversely related to the amplitude of the hare cycle (Fig. 2). This observation should be viewed in the current context not as an alternative but rather as a generalization including all the other mechanisms, each of which requires some environmental disturbance. In this particular case, we will use the generalized explanation of amplitude variation caused by the initial predator numbers, using lynx as a surrogate for all the predators that follow the hare cycle. Lynx are only one of the three major predators in the snowshoe hare cycle, but the population fluctuations of all three major predators are highly correlated (Krebs, Boutin & Boonstra, 2001). The data on hare abundance at Kluane Lake are shown in Fig. 1.

Lynx winter abundance in the Kluane region of the Yukon have been particularly high during the recent low of the hare cycle (14 tracks per 100 km of snow tracking in 2012), probably because of immigration into the study area, and from Fig. 2 we would predict the current hare cycle at Kluane Lake to have a low amplitude, with peak hare abundance of about 1 hare/ha or an amplitude of about 6 to be achieved in 2015 or 2016.

Our current hypothesis is that predator impact, while not affecting the period of the cycle, has a strong effect on the amplitude and thus on the very existence of the observed cyclicity. Because of the insensitivity of the period to the presence of predators, it is easy to not recognize this effect at all and claim predators to be complete “passengers” (White, 2013). The amplitude relation discovered by Krebs et al. (2014) points on the contrary to the definite role of predators in the very existence of cycles. After all, most species do not cycle in abundance and even the ones that do in one place may not do so in another. The very existence of cycles requires noticeable amplitude. Thus, complete “passengers” predators are not, although they may appear to be, due to the insensitivity of the cycle periods.

The most direct mechanistic argument in our favor is the observation of cyclic mammals continuing to show the attributes of the decline phase in the laboratory (in the absence of consumers) when sampled from the declining part of the cycle. This test has been shown clearly for some species of voles (Mihok & Boonstra, 1992; Nazarova & Evsikov, 2010) but not clearly for others (Ergon et al., 2001) and more recently has been observed for snowshoe hares (Sinclair et al., 2003; Sheriff, Krebs & Boonstra, 2009).

## Conclusion

In order to cycle one needs a time delayed, density dependent mechanism, or, equivalently, at least two-dimensional dynamics. This can be achieved by the traditional predator–prey models where abundances of the two species are the two dimensions but also by other two-dimensional models. Two dimensions, not necessarily two species, are required for cyclicity. The model based on the maternal effect and operating on generational time steps is not only a formidable competitor to the traditional predator–prey view, but may in fact be the preferred view. The eigenperiod hypothesis has substantial and multifaceted support in various data-based tests (Ginzburg & Colyvan, 2004). Krebs’ hypothesis on the amplitude of cycles of snowshoe hares being determined by the imbalance of predator abundance completes the picture. The two sets of evidence in combination reinforce the idea of the internally driven, second order dynamics of hare “pulled” by the predator imbalance away from the equilibrium. Just like in the mechanical analogy, the guitar string produces the same sound (eigenperiod) whenever it is pulled, but the loudness of the sound (the amplitude) depends on the strength of the pull.

A relatively weak but simple test of our proposed view about the cause of amplitude variation is our prediction of the very low amplitude of the current upcoming hare cycle in the Yukon. We will know the answer to this test by 2016. A more stringent test of our hypothesis will involve two strands. First, the generality of maternal effects will have to be tested on other mammal populations, and in particular the physiological and neurological mechanisms that underlie the maternal effects must be determined (Lavergne et al., 2014). The generality of maternal effects is critical to our hypothesis about the eigenperiod. Second, to test for amplitude effects we need a large-scale, multi-predator, multi-prey study on a landscape scale to test the simplest model of cyclic amplitude determination. Because of the mobility of the major predators in all these cyclic systems, measuring the survival and movements of predators is critical. With the advent of satellite telemetry, it is now possible to test our model directly.

We have addressed mammalian population cycles here, but we emphasize that this general model should also apply to birds and other vertebrates, as well as insect populations. The details of the physiological mechanisms will vary with different species but our general model, separating the factors generating population fluctuations from those affecting the amplitude of the fluctuations, should assist in experimental design and hypothesis testing.

## Additional Information and Declarations

### Data Availability

The following information was supplied regarding the deposition of related data: All data used in this paper are archived and freely available at the website http://www.zoology.ubc.ca/~krebs/kluane.html, and can be downloaded in an Excel file at http://www.zoology.ubc.ca/~krebs/downloads/monitor.xlsx.
